# Dissecting the development of the early lineages and primordial germ cells in the bovine embryo

**DOI:** 10.1590/1984-3143-AR2025-0098

**Published:** 2025-10-03

**Authors:** Anna Carolina Denicol, Ramon Cesar Botigelli

**Affiliations:** 1 Department of Animal Science, University of California Davis, Davis, CA, USA

**Keywords:** primordial germ cell, PGC, bovine, embryo, assisted reproduction

## Abstract

As cattle have not been traditionally considered a model species and the molecular details of germ cell development don’t directly inform production practices, the specification of primordial germ cells in the bovine embryo has remained understudied and poorly understood. Recent work by our laboratory builds on previous investigations to establish the molecular profile of primordial germ cells (PGC) at the critical moment when they are being specified in the embryo during the gastrulation stage. Combining advanced immunolocalization, confocal imaging and single-cell RNA sequencing, we identified PGC in the bovine embryo approximately on day 16 of development by co-expression of the core transcription factors OCT4, SOX17, PRDM1, and TFAP2C as demonstrated for several other species in which the embryo develops a bilaminar disc at the onset of gastrulation. Soon after specification, between days 20 and 22 of embryo development, early migratory PGC repress transcripts responsible for the establishment of somatic lineages. Notably, these cells do not seem highly proliferative during the early migratory stage, another aspect of early germ cells that is conserved in cows and other species such as pigs. Advancing the study of germ cell specification and development during bovine embryonic development, particularly at stages when human embryos are unavailable for investigation, places cows as an additional domestic species capable of providing crucial information about events that are paramount for fertility. As the field of in vitro gametogenesis continues to rapidly evolve, the study of bovine PGC and fetal germ cell development will provide invaluable information to facilitate the development and advancement of future assisted reproduction technologies for the improvement of agricultural animals and human reproduction.

## Introduction

The development of a multicellular organism, with all its complexities, from a one-cell zygote is arguably one of the most fascinating facets of biology. And the thing that makes it possible is the existence of germ cells. Germ cells, the origin of female and male gametes, appear very early during embryo development. These very special cells that will allow a species to continue to exist are specified during gastrulation while the embryo is undergoing significant waves of cell differentiation to establish the three main germ layers: ectoderm, mesoderm and endoderm.

Rodent models have been used for decades to understand events occurring during embryo development. Recently, however, there has been increasing interest in livestock species within the field of developmental biology. Recent advances including the establishment and improvement of pluripotent stem cells (PSC) and the availability of advanced molecular and genetic tools have fueled this interest and made it possible to return to these fundamental questions, which will in turn inform the steps necessary to advance assisted reproductive technologies and animal agriculture.

The aim of this review is to discuss the available information about the specification and initial development of primordial germ cells in cattle in light of what is known in other important large animal livestock species and animal models. Relevant contrasts will be presented as appropriate.

## Primordial germ cell specification in cows and other bilaminar disc embryos

At the onset of gastrulation, embryos from cows, sheep, pigs, rabbits, humans, and non-human primates develop into a structure called a bilaminar flat disc (Alberio et al., 2021). The dorsal aspect of the disk comprises the pluripotent epiblast, the cells that will give origin to primordial germ cells (PGC) while the ventral layer comprises the hypoblast. Initially, epiblast cells express the core pluripotency markers SOX2/POU5F1; however, as the embryo acquires antero-posterior patterning, cells in the posterior region of the epiblast lose expression of SOX2, and this marker remains expressed only in the anterior epiblast (Kobayashi et al., 2017).

Common between the bilaminar disc species better studied to date is the fact that PGCs originate in the posterior region of the epiblast (Alberio et al., 2021). PGC are specified as epiblast cells migrate ventro-medially and anteriorly within the forming primitive streak to become the mesoderm. The primitive streak is a hallmark structure of gastrulation in bilaminar disc structures that facilitates the migration of cells, including PGCs, within the embryo (Vejisted, 2010). In the pig, the closest species to cattle to be studied in great detail, PGC specification is marked by responsiveness of POU5F1+ epiblast cells to bone-morphogenic proteins, (BMPs), of which BMP4 seems to be the most critical (Kobayashi et al., 2017). In response to BMP4 and likely WNT signaling, these cells transiently express T (Brachyury) followed by the co-expression of the transcription factors SOX17, TFAP2C (AP2-gamma) and PRDM1 (BLIMP1) (Kobayashi et al., 2017). It is worth noting that although none of these proteins is exclusive of PGCs, their co-expression (POU5F1, SOX17, TFAP2C, and PRDM1) seems critical for repression of the somatic program and establishment of the germline. In vitro models of gastrulation using human embryonic stem cells have also elegantly demonstrated that acquisition of expression of the definitive endoderm marker SOX17 is the first event in PGC specification; next, SOX17+ cells must acquire PRDM1 expression to repress the endoderm fate during the transient window of PGC specification competency from mesendoderm-like cells before the establishment of the endoderm and mesoderm lineages (Irie et al., 2015; Kobayashi et al., 2017).

Before the establishment of a detailed molecular program of PGC specification in non-rodent species, Wrobel and Süss (1998) examined cattle embryos between days 18 and 39 of development and established the presence of PGC at day 18 by positive alkaline phosphatase activity and lectin staining. Although this was a pioneer report on the identification of PGCs, the marker used was not germ-cell specific and questions still remained as to whether this was the actual time of PGC specification. Primordial germ cells were not reported in a comprehensive analysis of bovine embryos at day 11 and 14 (Maddox-Hyttel et al., 2003), and in that report, alkaline phosphatase activity was detected in trophoblast and hypoblast cells.

Given that PGCs are specified in the posterior epiblast or nascent amnion in bilaminar disc embryos, and since these structures, as well as the pre-primitive streak structure, are present at day 14 in the bovine embryo, we investigated if PGCs could be found two days later, at day 16 of embryo development. We took advantage of the knowledge generated in mouse, pig and rabbit embryos and utilized specific markers of the embryonic lineages developing at that point (Saitou et al., 2002; Ohinata et al., 2005; Kobayashi et al., 2017, 2021). At day 16 of development, bovine embryos are at the early gastrulation stage, and the embryonic disc is clearly formed by a well-developed epiblast (POU5F1+) underlined by a layer of hypoblast cells (SOX17+/PRDM1+). This structure is surrounded by the trophoblast (TFAP2C+). Notably, bovine embryos at day 16 had clearly established an antero-posterior pattern of the pluripotent epiblast marked by mutually exclusive expression of SOX2 (anterior) and TBXT (posterior, nascent mesoderm). We observed a thickening in the posterior region between the epiblast and the hypoblast as described by Maddox-Hyttel et al. (2003) in day 14 bovine embryos which corresponded to the forming primitive streak. Within this region and close to the trophoblast, cells co-expressing POU5F1, SOX17, TFAP2C, and PRDM1 were identified (Guiltinan et al., 2025). Although the number of embryos we examined to date is limited (n = 6), the presence of core transcription factors of PGC specification as reported in other bilaminar disc embryos allowed us to identify these cells as PGC in the bovine embryo.

**Figure 1:**
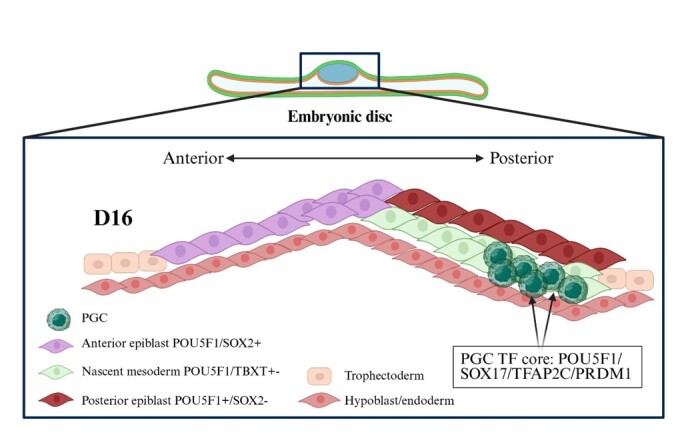
Model of primordial germ cell (PGC) specification in the bovine embryo during early stages of gastrulation. PGC: primordial germ cell; TF: transcription factor; D16: day 16 of embryonic development.

We conclude from these observations that in bovine embryos, PGCs are likely specified in the posterior epiblast approximately at day 16 of development by activation of expression of SOX17, TFAP2C and PRDM1 as described in other bilaminar disc-forming embryos (Figure 1).

## Fate of (female) germ cells after specification

At the time PGCs are specified, the embryo is undergoing rapid changes as the main germ layers – endoderm, mesoderm and ectoderm – are being established. Five days after specification, the embryo is undergoing rapid differentiation and somitogenesis has occurred (Maddox-Hyttel et al., 2003). The appearance of a well-delineated neural tube marks neurulation (Maddox-Hyttel et al., 2003), and the segregation of the anterior epiblast has originated a large region of neural ectoderm cells that are SOX2+/POU5F1+ (Guiltinan et al., 2025). In the posterior region of the embryo, POU5F1 has become exclusive of the few cells that remain pluripotent in the embryo: the PGCs. Primordial germ cells can be localized to the hindgut/early allantois by the co-localization of POU5F1, PRDM1, SOX17 and TFAP2C (Guiltinan et al., 2025), demonstrating early migratory stages. As previously demonstrated in pig embryos (Zhu et al., 2021), bovine PGCs at this stage do not seem highly proliferative based on cell cycle analysis and low prevalence of Ki67 protein expression (Guiltinan et al., 2025). This could indicate that the recently specified bovine PGCs are not proliferative, as demonstrated in mice and pigs (Seki et al., 2007; Kobayashi et al., 2017), but remains to be fully elucidated.

## Primordial germ cell-like cells (PGCLC) as in vitro-produced PGC counterparts

The difficult access to precious human embryos has fueled a significant effort to develop in vitro models of embryonic development. Of relevance to this review, recent reports demonstrate the formation of human gastruloids where PGC specification occurs spontaneously in response to endogenous signaling from neighboring cells as occurs in the embryo proper (Cooke et al., 2023; Neupane et al., 2025). Although gastruloids made of pluripotent stem cells undergo extensive characterization to ensure that they are closely replicating natural development, these models can only go so far at explaining complex developmental processes.

Similarly, the induction of PGCLC from pluripotent stem cells in 2D or 3D systems, but without the use of blastoid or gastruloid models, has advanced significantly in non-rodent species, although functional validations are yet to be reported. In the mouse, the complete oogenic cycle was demonstrated by the seminal work of Katsuhiko Hayashi’s group in 2016 (Hikabe et al., 2016). In that report, the authors showed that pluripotent stem cells could be induced into PGC-like cells after a brief differentiation into epiblast-like cells (necessary since mouse PSC are typically in the naïve state of pluripotency). Once induced, PGCLC were sorted based on endogenous expression of PRDM1 through reporter systems, and assembled into reconstructed ovaries by combination with ovarian somatic cells from mouse embryos. In these reconstructed ovaries, PGCLC and somatic cells assembled in follicles that developed to the antral stage and were subjected to in vitro maturation, fertilization, and embryo transfer. The resulting pups had normal birth body and placental weight and grew up to be fertile, producing offspring. These pups were also used as a source of embryos from which embryonic stem cells were isolated and put through the entire second round of in vitro oogenesis (Hikabe et al., 2016). At the time of writing this manuscript, PGCLC induction in non-rodents has been reported in humans (Esfahani et al., 2024), cattle (Shirasawa et al., 2024), rabbits (Kobayashi et al., 2021), pigs (Gao et al., 2019), and rhinoceros (Zywitza et al., 2022). However, no functional characterization such as successful fertilization of an in vitro-produced oocyte has been reported. In fact, no oocytes have been reported yet in any of these species. Table 1 summarizes the full reports currently available in the literature about PGCLC induction from bovine embryonic stem cells.

**Table 1:** Peer-reviewed, full-length reports of primordial germ cell-like cell (PGCLC) induction from bovine embryonic stem cells (ESC)*.

**ESC source**	**Pre-differentiation (yes/no) and method**	**PGCLC differentiation method and medium**	**PGCLC markers**	**Reference**
Expanded potential mTeSR 1, + 1 µM CHIR99021, + 0.3 µM WH-4-023, +.5 µM IWR1, + 10ng/mL rhLIF, + 20ng/mL Act A, + 50µg/mL Vit C	2D Seeded on gelatinized plates in M15 medium, + 10 µM ROCKi, + 20 µg/ml Act, + 2.0 µg/ml Dex SOX17 overexpression (piggyBac based PB-CAG-SOX17-GR expression construct) 12 hours	3D 5,000-6,000 cells per aggregate Advanced RPMI 1640, 1% B27, 500ng/mL BMP2, 10ng/mL hLIF, 100ng/mL SCF, 50ng/mL EGF, and 10 μM ROCKi (Y-27632) 3-4 days	no described results.	Zhao et al. (2021)
Primed N2B27 base, + 2.5 μM IWR1, + 10ng/mL Act A, + 20ng/mL FGF2	2D 2-3x10^5 cells in 12WP coated with fibronectin N2B27 base, + 5% KSR, + 100ng/mL BMP4, + 6µM CHIR, + 2.5µM IWR, + 10µM ROCKi 24 hours	3D 6,000 cells per aggregate GMEM, 15% KSR, 200ng/mL BMP4, 10ng/mL LIF, 100ng/mL SCF, 50ng/mL EGF, 6µM CHIR, 2.5uM IWR, and 10µM ROCKi 4 days	Flow Cytometry: TFAP2C+/PRDM1+ reporters bulk RNAseq: Sorted PGCLC vs ESC IF: PRDM1, TFAP2C, SOX17, NANOG, POU5F1, SOX2- H3K27me3, H3K9me2, 5mC	Shirasawa et al. (2024)
Formative - 3iLAF N2B27 base + 5% KSR, + 1 µM CHIR99021 + 0.5 µM IWR1, + 1 µM WH-4-023, + 10ng/mL rhLIF, + 25ng/mL Act A, + 10ng/mL FGF2	3D - 3,000 cells 3iLAF bESC medium 24 hours	3D N2B27, 5% KSR, 200ng/mL BMP4, 10ng/mL rhLIF, 100ng/mL rhSCF, 50ng/mL EGF, and 10µM ROCKi 3 days	RT-qPCR: PGCLC versus bEpiSCs Upregulation of TFAP2C and PRDM1	Zhi et al. (2024)
Formative - JY mTeSR-plus +2.5 µM IWR1 + 3.3 µM iDOT1L + 0.8 µM PD184352 + 2 µM SU5402 + 3 µM CHIR99021 + 10 µM Forskolin + 1,000 U/ml hLIF	No pre-differentiation.	3D 3,500 cells per aggregate GMEM, 15% KSR, 500ng/mL BMP4, -/+ 500ng/mL BMP8a, 1000 U/mL LIF, 100ng/mL SCF, 50ng/mL EGF, and 10µM ROCKi 4 days	IF: TFAP2C, PRDM1, SOX17, H3K27me3, and H3K9me2 +BMP8a increased expression of TFAP2C, PRDM1, KIT, DPPA3, and TDRD5	Su et al. (2024)

* Abstracts and conference proceedings are not included in this table.

## Applications of basic knowledge to develop novel assisted reproductive technologies

The development of new assisted reproductive technologies relies on knowledge of basic reproductive biology within a species, extrapolation of basic knowledge from other species, or more often a combination of both. The field of in vitro gametogenesis from stem cells has grown significantly since the publication of pluripotent stem cell-derived spermatids (Hayashi et al., 2011) and oocytes (Hikabe et al., 2016). Although the originally published protocols have repeatedly demonstrated success in mice, to this date a functional gamete has yet to be produced from stem cells in any other species. This could be largely attributed to the “immaturity” of the stem cell field in livestock and other domestic animals compared to mice (Botigelli et al., 2023) and also to the lack of detailed molecular development studies in those species. As a classic example, PGC specification remained elusive in non-mouse species due to the lack of SOX2 expression, when in the mouse this transcription factor is known to be required in nascent PGCs (Maddox-Hyttel et al., 2003). The fact that mouse embryos develop as an egg cylinder at the onset of gastrulation, as opposed to the bilaminar disc structure found in pigs, rabbits, humans, and cows, helps inform this and other discrepancies that have come to light. This highlights the critical need for a comparative approach to the study of developmental biology, as clearly not all embryos are made the same. Our recent studies of bovine PGC specification and migration have shed light into the similarities and also differences between cows and other species where this information is known (Guiltinan et al., 2025). For example, induction of PGC-like cells from bovine pluripotent stem cells should yield cells that are SOX17+/OCT4+/PRDM1+/TFAP2C+, but SOX2-. If those conditions are not met, the produced cells would be unlikely to form functional gametes. Although seemingly trivial, this is fundamental knowledge that until very recently was not known.

## Conclusion remarks

The generation of basic knowledge about fundamental developmental processes, namely primordial germ cell specification, in non-traditional model species such as cattle has profound implications to the successful development of advanced assisted reproduction technologies. As a clear example, the exploitation of the idea of in vitro gametogenesis could lead to the development of strategies to perform in vitro breeding, accelerating the rate of genetic gain while reducing the environmental impact of producing animals in the process (Goszczynski et al., 2019; Botigelli et al., 2023).

The finding that PGC specification in bovine embryos follows closely what occurs in other bilaminar disc embryos such as pigs and humans highlights the conservation of this phenomenon in species that undergo similar gastrulation development and reinforces the critical importance of utilizing the best available model species to answer specific questions in developmental biology. The mono-ovulatory nature of cows, the easy access to bovine ovaries, semen, and highly efficient methods for embryo production in vitro and embryo transfer, the availability of advanced molecular tools such as CRISPR/Cas9, combined with the delayed implantation of bovine embryos in the uterus, makes the cow a very desirable and more importantly, a feasible model to study PGC specification and development.

Beyond the use of cows as a model species, the critical economic and social roles of cattle production worldwide make the elucidation of basic developmental milestones valuable for enabling future technologies and field applications.

## Data Availability

No research data was used.
